# An Acute Hyperoxia Test Predicts Survival in Children with Pulmonary Hypertension Living at High Altitude

**DOI:** 10.1089/ham.2021.0026

**Published:** 2021-12-13

**Authors:** Gabriel F. Diaz, Alicia Marquez, Ariel Ruiz-Parra, Maurice Beghetti, Dunbar Ivy

**Affiliations:** ^1^ Department of Pediatrics, Universidad Nacional de Colombia, Fundación Santa Fe de Bogotá, Bogotá Colombia.; ^2^ Clínica De La Mujer, Centro Policlínico del Olaya, Bogotá, Colombia.; ^3^ Instituto de Investigaciones Clínicas and Department of Obstetrics and Gynecology, Universidad Nacional de Colombia, Bogotá, Colombia.; ^4^ Head of Pediatric Cardiology Unit (HUG), Director Pulmonary Hypertension Program (HUG) Children's University Hospital, Geneva, Switzerland.; ^5^ Chief and Selby's Chair of Pediatric Cardiology, University of Colorado, School of Medicine, Children's Hospital Colorado, Denver, Colorado, USA.

**Keywords:** early detection of pulmonary hypertension, high altitude, pulmonary hypertension in children, hyperreactivity of pulmonary vasculature, hypobaric hypoxia, O_2_Test

## Abstract

Diaz, Gabriel F., Alicia Marquez, Ariel Ruiz-Parra, Maurice Beghetti, and Dunbar Ivy. An acute hyperoxia test predicts survival in children with pulmonary hypertension living at high altitude. *High Alt Med Biol*. 22:395–405, 2021.

***Background:*** Pulmonary hypertension (PH) causes significant morbidity and mortality in children at altitude.

***Materials and Methods:*** Fifty-two children living at 2,640 m were included. During hyperoxia test (O_2_Test), patients received high oxygen concentrations (FiO_2_ >80, through Mask, using Venturi or nonrebreathing mask); echocardiography was used to evaluate pulmonary vasculature reactivity. A decrease >20% from the basal pulmonary artery systolic pressure was considered a positive response.

***Results:*** Most of the patients had severe PH. The median age at diagnosis was 4.5 years; 34 were female (65.4%). Idiopathic PH was present in 44 patients (84.6%). Six developed severe PH after ductus closure. They were classified in responders (*n* = 25), and nonresponders (*n* = 26). Responders were younger (3 years vs. 7 years, *p* = 0.02), and 22 (88%), had better functional class (FC) 1–2, than nonresponders: 18 (69.23%) of them had worse FC: 3–4 (*p* = 0.000). In responders, 10/12 who went to live at low altitude became asymptomatic, compared with 7/13 who remained at high altitude. FC 1–2 was achieved by 70% of the patients with idiopathic PH who went to a low altitude, compared with 30% who continued at high altitude (*p* = 0.03). In nonresponders, 10/26 patients moved to a low altitude: four improved, one worsened, and five died; of the 16/26 patients living at high altitude, four are stable, eight worsened, and four died. Four patients (30.76%) in responder group and nine (69.24%) in the nonresponder group died (*p* = 0.03). There were differences between both groups in systolic (88 mm Hg vs. 110 mm Hg; *p* = 0.037), diastolic (37 mm Hg vs. 56 mm Hg; *p* = 0.035), and mean pulmonary artery pressures (57 mm Hg vs. 88 mm Hg; *p* = 0.038).

***Conclusions:*** This specific hyperoxia test applied until 24 hours (not published before) helps to predict survival and prognosis of children with PH. Children with PH at a high altitude improve at low altitude.

## Introduction

There is scarce information about pulmonary hypertension (PH) in children living at high altitudes (Rajagopal et al., 2016). Children with PH living at high altitude reveal special characteristics, as different to children with PH living at sea level or near sea level, the hypobaric hypoxia at altitude may lead to aggravation of PH due to hypoxic pulmonary vasoconstriction (HPV), and these children may reveal special vasoreactivity to high fraction of inspired oxygen (FiO_2_) (Díaz and Márquez, 2011; Peñaloza, 2011).

Due to these unique traits, we suggest a new diagnostic and therapeutic approach. The pediatric classification of PH (Panama Classification) (Del Cerro et al., 2011) identifies pediatric PH related with hypobaric hypoxic exposure (hypoxic PH syndrome) (Tianyi, 2007) in a separate group; the Nice World Symposium calls out chronic exposure to high altitude as a risk factor for PH (Galiè et al., 2019; Rosenzweig et al., 2019; Simonneau et al., 2019).

Children with PH who live at high altitudes present differently compared with children with PH living at low altitudes or at sea level because of the effect of hypobaric hypoxia. Hypobaric hypoxia is defined as decreased oxygen availability, secondary to low partial pressure of oxygen at high altitudes. It is known that as the altitude above sea level increases, the barometric pressure, the partial pressure of oxygen (*PO_2_*), *P_A_O_2_*, *P_a_O_2_*, and oxygen saturation decrease (Montes de Oca Sandoval et al., 2010).

The effects of hypobaric hypoxia on the physiology and physiopathology of PH in children living at high altitudes are observed from birth and possibly from intrauterine life if the mother is exposed to hypobaric hypoxia (Soifer et al., 1983; Rood et al., 2019). This explains the slower decrease of pulmonary resistances after birth at a high altitude compared with its rate of decline at sea level (Sime et al., 1963; Vogel et al., 1964; Gamboa and Marticorena, 1971; Niermeyer, 2003).

Furthermore, hypobaric hypoxia increases the vasoconstriction response and remodeling of the pulmonary vasculature (Wagenvoort and Wagenwoort, 1973; Rhodes, 2005; Penaloza and Aras Strohl, 2007; Díaz and Márquez, 2011; Sylvester et al., 2012; Mirrakhimov and Strohl, 2016; Dunham-Snary et al., 2017; Neupane and Swenson, 2017). Vasoconstriction related to hypoxia can be either regional in specific areas or generalized affecting the whole pulmonary vasculature as it occurs at higher altitudes (Stenmark et al., 2006).

According to Peñaloza et al. (1964), the effect of hypobaric hypoxia on pulmonary pressure at high altitudes (above 2,500 m) follows a fast-rising parabolic curve. Additionally, prolonged exposure to hypobaric hypoxia in susceptible people, such as patients with PH, can lead to irreversible remodeling structural changes, which are the final stage and should be avoided.

*Objectives:* The main objective of this study is to present the usefulness of a prolonged hyperoxia test (O_2_Test) to identify patients who still have a reactive pulmonary vasculature. When the O_2_Test is positive, the prognosis is better, correlating with the Functional Class (FC).

## Patients and Methods

The study was approved by the ethical committees of the National University of Colombia and the participant institutions. All parents of the patients signed an informed consent for the O_2_Test.

This is a prospective study of children diagnosed with PH who were born and lived in Bogotá, Colombia, at 2,640 m, which is considered high altitude (Parati et al., 2018), as well, as two additional patients who were born at low altitude, but were diagnosed with PH in Bogotá.

This study included children from our database who had significant PH and continuous follow-up (1983–2020) had been done. Most of the patients had severe PH, defined as pulmonary pressure near systemic level, at a systemic level, or at a suprasystemic level, during cardiac catheterization. All patients underwent a meticulous clinical evaluation, an EKG, a chest X-ray, an echocardiogram, a heart catheterization, and a prolonged hyperoxia test.

**FIG. 1.**
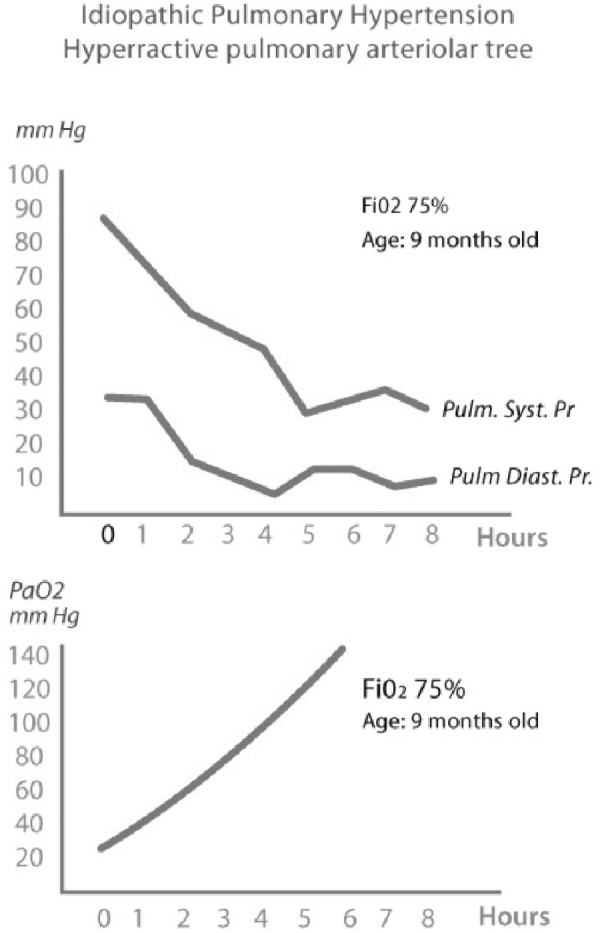
Graphics demonstrating the results of the Prolonged Hyperoxia Test in a 9-month-old patient with suprasystemic pulmonary pressure and 17 WU Res. (37 years ago) with doubt of vascular reactivity test in the catheterization. In the superior panel, it is shown that the systolic and diastolic pulmonary pressures went down progressively until normal after 6 hours of hyperoxia. In the inferior panel, it is shown that the PaO_2_ went up, from 30 to 140 mm Hg after 6 hours of hyperoxia. The catheter was left in the pulmonary trunk to measure the pulmonary pressure.

This study began 37 years ago on a 9-month-old infant with severe PH, in whom there was doubt about the reactivity of the pulmonary vasculature during the catheterization (Fig. 1). As a result of this study with FiO_2_ >0.75, one of the authors (GD) began to apply the O_2_Test using echocardiography to children with PH living at high altitudes. The O_2_Test consists of a supplementary supply of oxygen by mask (nonrebreather mask or Venturi) at FiO_2_ >0.8 (value chosen, taking into account the results of our first cases) for at least 4 hours and a maximum of 24 hours continuously, except for feeding and drinking. Echocardiographic measurements were made before and after the hyperoxia exposure.

Pulmonary artery systolic pressure (PASP) was estimated by echocardiography through the tricuspid regurgitation jet velocity using the Bernoulli equation, plus the estimated right atrial pressure. Taking into account the severity of pulmonary pressure, we added 10 mm Hg to the regurgitant gradient. When pulmonary regurgitation was present, pulmonary diastolic pressure was estimated through the same principle. The presence of foramen ovale or atrial defect was considered. In last patients, other parameters such as the right ventricle (RV) and right atrium size, RV/LV (left ventricle) rate, LV eccentricity index, TAPSE, and IVC collapsibility were evaluated. Initially, the studies were made with a General Electric Vivid I and later with General Electric Vivid Q echocardiographs. From each patient, informed consent for the O_2_Test was obtained.

A positive response to the O_2_Test was defined as a decrease more than 20% from the basal PASP without any change in the systemic blood pressure, according to the finding and outcome of the first cases. This definition was a modification of the Barst criteria for reactivity. The first evaluation was made at 4 hours of hyperoxia, and, if there was no response, the hyperoxia was prolonged 24 hours. The systemic blood pressure was measured simultaneously. All echocardiographic studies were made by two experts in echocardiography in children with PH (A.M. and G.F.D.). It was necessary to get at least four excellent and repetitive evaluations at the time of the echocardiography to obtain a good correlation with the catheterization findings.

If response to the O_2_Test was positive or negative, we recommended to the patients to move to a low altitude. For the first patients, this was the only treatment. From 2002, we began to use sildenafil, and later sildenafil plus bosentan or macitentan in each patient, according to the clinical situation, without taking into account if the patient could go or not to live at a low altitude.

Statistical analysis was performed with Stata version 15.0 (Texas, USA). The variable distribution was analyzed using the Shapiro–Wilk test. Data are shown as median and percentiles when distribution was not normal and as mean and standard deviations when the distribution of the data was normal; in both cases, the ranges are shown. The Student's *t*-test was used for comparison of normally distributed data, the Mann–Whitney test for comparison of data not distributed normally, and the Chi-square test for comparison of proportions. Kaplan–Meier functions were estimated for survival analysis.

## Results

### Basal population

Fifty-two patients were included in this study (Table 1). The median age at diagnosis was 4.5 years [interquartile range; IQR: 2–10 years]; 23 (44.2%) were diagnosed at three years old or younger; 45 children were born at term and only one was born below 30 weeks of gestation; 34 were female (65.4%). Idiopathic PH was present in 44 patients (84.6%). Six developed severe PH after patent ductus closure (two of them with Down syndrome). For other associated pathologies see Table 1.

**Table 1.** The Main Characteristics of the 52 Patients

Patient	Age at diagnosis	Dx	PSP 1	PHT	PSP 2	Follow-up	Outcome	Low altitude	Treatment	Year of entry	Catheterism and VRT: O_2_—No	Pulmonary resistance	FC	Catheterism 2	Resistances	Pregnancy W	FO
Female	9 Months	IPH	97	Yes (+)	37	6 Months	Dead	NO	Oxygen	1983	97/38 56	17 UW	3			40	Yes
Female	2 Years	IPH	94	Yes (+)	64	37 Years	Good	Yes	Oxygen	1983	92/45 66	14 UW	2			40	No
Male	5 Years	IPH	102	Yes (+)	68	37 Years	Very good	Yes	Oxygen	1983	102/58 78	19.3 UW	1			40	No
Female	4 Years	IPH	98	Yes (−)	96	1 Year	Dead	Yes	Sildenafil +O_2_	2008	110/52 84	23 UW	4			40	No
Female	7 Years	VSD Eisenmenger	102	Yes (−)	98	13 Years	Stable	Yes	Sild + Bosentan	2007	97/59 75	8.8 UW	3			40	No
Male	2 Years	IPH, assoc. VSD	119	YES (−)	110	13 Years	Stable	Yes	Sild −Bosentan	2007	133/55 90	20 UW	2			40	No
Male	7 Years	IPH	160	YES (+)	120	2 Years	Dead	No	Sildenafil	2010	160/110 138	32.4	4			40	No
Female	1.5 Years	PH, VSD	86	YES (+)	62	16 Years	Good	Yes	Sild + Cirugía	2004	88/36 58	10.4 UW	2			40	No
Male	21 Months	IPH	153	YES (+)	94	18.5 Years	Very good	Yes	CA+ CH BL^*^	2002	84/20- 48	12 UW	1	54/16,32; 45/15,23; 49/14, 25	3.62,0.67,1.18	38	No
Female	1 Years	IPH	145	YES (+)	70	3 Years	Dead, different cause	Yes	Sild Bosentan	2011	136/67 97	23.62 UW	2			40	Yes
Female	2 Years	IPH	112	YES (+)	40	11 Years	Dead, pneumonia	Yes	Sild + Bosentan	2005	114/78 96	28.67	2			40	No
Female	11 Months	IPH	111	YES (+)	58	11 Years	Very good	Yes	Sild + Bosentan	2009	76/35 56	7.3UW	1			40	No
Female	7 Years	IPH	170	YES(−)	168	6 Months	Dead	No	Sild + Bosentan	2002	104/62 88	24.6	4			40	No
Female	2.5 Years	IPH	104	YES (−)	89	2.5 Years	Dead	Yes	Sild + Bosentan	2010	110/78 92	26.4	3			40	No
Male	8 Years	IPH	80	YES (−)	56	7 Years	Dead in taekwondo	Yes	Sild + Bosentan	2011	72/35 55	10.65 UW	3			40	No
Male	3 Years	IPH Ductus Closure	140	Yes (−)	132	4 Years	Dead	Yes	Sild + Bosentan	2013	145/87 109; 127/73,95; 130/75,96	78.6; 39.33; 39.81	3			40	Yes 2.8 mm
Male	6 Months	CIV Ductus Eisenmenger	78	Yes (−)	68	37 Years	Worse	No	Sild + Macitentan	2003	70/37 50	23.4 UW	3			40	Yes
Female	13 Years	IPH	92	Yes (+)	42	7 Years	Stable good	No	Sild + Bosentan	2013	88/37/57	8.28	2			40	No
Female	12 Years	IPH	98	Yes (−)	92	4 Years	Dead	Yes	Sild − Macitentan	2014	90/43 63; 86/31,51; 78/35,51	18.46; 14.20; 12.73	3	90/39,60; 86/31,61; 76/35,51	18.84; 14.20; 12.73	40	No
Female	6 Years	IPH Ductus Closure	96	Yes (−)	78	5 Years	Worse- higher PP	No	Sild Bosentan	2015	73/36 55	16.94	3			40	No
Female	17 Days	PAH CIA CIV ductus	84	Yes (+)	42	7 Years	Good	Yes	Cirugia	2013	48/22 16	2.4	1			40	ASD
Female	1 Year	IPH	128	Yes (+)	78	5 Years	Good	No	Sild + Bosentan	2015	114/51, 77; 88/34,54; 98/32,58	38.78; 26,02; 31.28	1			40	No
Female	6 Years	IPH	118	Yes (−)	102	6 Years	Dead	No	Sild Bosentan	2014	137/55,89; 124/43,76; 106/42 66	38.41; 25.16; 25.41	3b			40	No
Male	13 Years	PH after ductus closure	144	Yes (−)	136	3 Years	Dead	No	Sild; bos trep^**^	2013	139/63 83	28.55	4			36	No
Female	13 Years	IPH	130	Yes (−)	128	2 Years	Dead	No	Sild, bos trep	2016	126/51 83; 122/57,64; 123/52,81	15.27; 10.74; 14.61	4			40	Yes
Female	10 Years	IPH	92	Yes (+)	42	14 Years	Very good	Yes	Nothing	2006	81/33 53	12.2	1			40	No
Male	5 Years	IPH	128	Yes (−)	122	2 Years	Dead	No	Sild Bosentan	2016	122/56 90; no change with O_2_—no	33.13	4			40	No
Female	2 Years	IPH		Yes. No tr reg		5 Years	Stable	No	Sild + Bosentan	2015	124/67,97; 114/51, 77; 98/32, 58 .-	43.57; 38.78; 26.02	2	111/53,81; 106/53,69; 100/34,65	24.81;25.19; 18.02	36	Yes 5 mm
Female	9 Years	IPH	113	Yes (−)	108	2 Years	Bad worse	No	Sild + Bosentan	2018	141/71,98; 126/61,82; 129/58/85	44.72; 26.26; 31.84	3			38	No
Female	1 Year	IPH	94	Yes (−)	86	5 Years	Stable regular	No	Sild + Bosentan	2015	69/30, 46; 68/17,42; 68/29,46	30.93; 21.83; 22.95	2			40	No
Female	3 Years	IPH + small VSD	145	Yes (−)	138	4.5 Years	Stable	Yes	Sild + Bosentan	2016	127/67, 93 no changes to O_2_	37.64; no changes	2			41	ASD 8 mm,
Female	14 Years	IPH	108	Yes(−)	98	3 Years	Stable regular	No	Sild + Bosentan	2017	110/54, 73; 118/55, 78; 88/46,62	24.4; 25.7; 22.63	2			36	No
Female	2 Years	IPH + FO 4 MM	115	Yes (+)	85	4 Years	Regular	No	Sild + Bosentan	2016	119/72,91; 116/44,76; 106/39,69	50.46; 41.06; 35.26	2			40	Yes
Female	5 Years	IPH	99	Yes (−)	82	3 Years	Bad	No	Sild Bosentan	2017	110/69, 85; 107/60, 80; 94/58, 72	47.22; 32.8; 24.76	3			40	No
Female	2 Years	IPH POP Ductus	63	Yes (+)	46	4 Years	Excellent PSP 33 mm	Yes	Sild	2016	88/33,61; 76/24, 49	43.0; 19.26	2			27	Yes fo 3 mm
Female	3 Months dow	PH POP of AVSD	68	Yes (+)	38	5 Years	Stable	No	Nothing	2015	43/14, 28	3.3	2			35	AVSD
Female	10 Years	PH + FO. ASMA GR3	79	Yes (+)	56	5 Years	Stable regular	No	Nothing	2015	68/33, 50; 49/16, 30; 49/17, 30	11.19; 4.3; 4.08	2			39	Yes 3.6 mm
Female	12 Years	IPH Down	73	Yes (−)	68	4 Years	Stable regular	No	Sild + Bosentan	2016	58/!4,36; 61/17,37	8.6; 8.52	2			40	Yes
Male	4 Years	IPH	162	Yes (−)	148	4 Years	Worse-higher PP	No	Sild + Bosentan	2016	101/39, 69; 99/31, 70	11.24; 10.22	2			40	ASD 10 mm
Female	15 Years	PH + Diaph Hernia	115	Yes (−)	112	2 Years	Worse	No	Sild + Bosentan	2018	88/41,64; 80/36,57; 77/32,53	7.07; 4.4; 6.03	3			40	Yes restr dvpap
Male	14 Years	Eisenmenger	104	Yes (−)	98	6 Years	Stable regular	No	Sild + Bosentan	2014	133/64, 87	19.6	2			40	No
Female	12 Years	IPH	105	Yes (−)	96	4 Years	Died	No	Sild + Bosentan	2016	92/47, 67; 82/39, 56; 70/37,50	9.54; 6.11; 5.15	2			40	No
Male	10 Years	IPH	74	Yes (+)	58	2 Years	Very good	Yes	Sild + Bosentan	2018	58/15,31;48/16,326; 39/16,23	4.31; 1.29, 1.6	1			40	No
Male	1.5 Years	IPH + ASD	98	Yes (+)	66	2 Years	Good	No	Sild + Bosentan	2018	72/35 55; 49/24,32	13.7; 4.66	2			40	ASD 10 mm
Female	2 Years	IPH	60	Yes (+)	38	1 Year	Good	No	Sild	2019	88/23, 55; 68/21, 41; 48/13, 27	10.6; 3.3; 1.5	1			40	No
Female	12 Years	IPH	118	Yes (−)	110	4 Years	Bad	Yes	Sild + Macitentan	2016	109/70, 86; 95/59, 75; 197/63, 81	41.18, 19.87, 33.38	3-Jan			40	No
Female	2 Years	IPH	88	Yes (+)	62	5 Years	Bad	No	Sild Bosentan	2015	127/68,93; 112/47,75	24.81; 26.30	3			36	No
Male	12 Years	IPH Hape	53	Yes (+)	42	4 Years	Very good	No	Nothing	2016	51/20, 34; 45/19,31; 51/22, 35	6.37, 5.65; 5.35	1			37	No
Male	14 Years	PH Pneumonia G3	70	Yes (+)	44	4 Years	Good	No	Nothing	2016	67/27,42; 57/17,34; 53/21,33	13.39; 5.09	2-Jan			35	No
Male	3 Years	IPH + Assoc ASD	73	Yes (+)	58	4 Years	Excellent	Yes	Sild + Bosentan	2016	103/58, 80; 86/43, 62; 88/38, 61	20.9; 24.8; 12.09	1			38	Yes
Male	4 Years	IPH	120	Yes (+)	64	3 Years	Very good	No	Nothing	2017	120/61,90; 71/18,39; 60/21,36	57,39; 22,94; 6.19	1			40	No
Female	8 Years	IPH POP—Ductus	72	Yes (−)	62	2 Years	Regular	No	Sild + Bosentan	2018	103/67,83; 102/55;76; 101/57,76	31, 13, 24	3			40	No

^*^ Calcium channel blockers.

^**^ bos trep: bosentan, treprostinil.

ASD, atrial septal defect; FC, functional class; IPH, idiopathic pulmonary hypertension; PH, pulmonary hypertension; PHT, prolonged hyperoxia test; PSP, pulmonary systolic pressure; VSD, ventricular septal defect; VRT, vascular reactivity test.

**FIG. 2.**
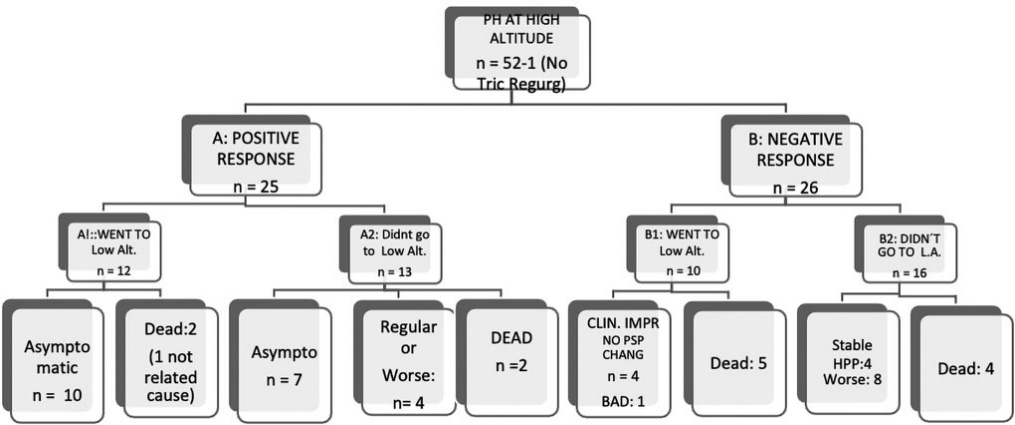
Flow chart of patients with pulmonary hypertension, showing the outcome, according to, if they went or not to live at low altitude.

Regarding the O_2_Test response, the patients were classified into two groups: group A, responders, or positive to O_2_Test (*n* = 25, 49.02%); and group B, nonresponders or negative to O_2_Test (*n* = 26, 50.98%) (Fig. 2). One patient without tricuspid regurgitation did not have O_2_Test. Patients in group A were younger at diagnosis than patients in group B (median 3 years old vs. 7 years old, *p* = 0.02). Twenty-two of 25 patients (88%) in group A had Panama FC 1 or 2 while in group B, 18 of 26 (69.23%) had FC 3 or 4 (*p* = 0.000).

### Clinical outcomes

Subgroup A1 (*n* = 12) corresponds to patients with a positive response to the O_2_Test who went to live at low altitude; 10 of these patients became completely asymptomatic, and two died (one death was not related to PH). In most of the patients, the PASP went down after living at low altitude and receiving vasodilator therapy. In subgroup A2, which corresponds to positive responders who remained at high altitude (*n* = 13), seven became asymptomatic (six with mild PH), four are in moderate condition or have gotten worse with deterioration in the FC and PASP, and two patients died (Fig. 2).

Regarding the group of Idiopathic PAH, when the patients went to live at low altitude, 70% (*n* = 10) achieved a better FC (1 or 2), whereas, of the patients that stayed at a high altitude, only 30% (*n* = 2) were in FC 1 or 2 (*p* = 0.03). The FC and the quality of life were better in patients that could go to live at a low altitude.

**FIG. 3.**
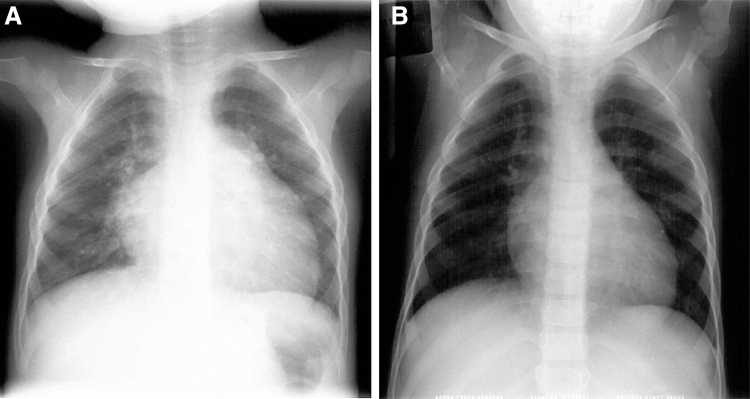
Thorax X-ray of patient 9, table 1, comparing the initial findings (21 months old, with suprasystemic PP, positive answer to the O_2_Test, and hyperreactivity of pulmonary vasculature), with current findings (19 years old). **(A)** Initial thorax X-ray: important cardiomegaly with enlargement of right atrium and right ventricle and important dilation of the pulmonary trunk and central branches. **(B)** current thorax X-ray after 17 years of follow-up (FC 1): There is mild enlargement of the right atrium and right ventricle. FC, functional class.

**FIG. 4.**
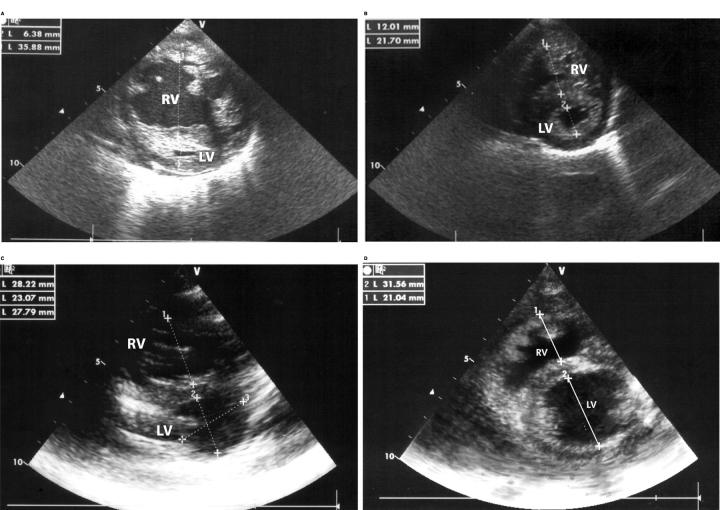
Patient 22 in Table 1, initially in FC 3, suprasystemic PH and 38.7 WU Resistances, with positive answer to the O2Test and hyperreactivity of pulmonary vasculature. **(A, B)** Initial Echo (1 years old). Short-axis view of the ventricles in Systole **(A)** and Diastole **(B)**. These views show a very small left ventricle mainly in Systole. **(C)** (Systole) and **(D)** (Diastole): Current echocardiogram. Short axis view of the ventricles 6 years after treatment and in FC 1. The diameter of the left ventricle is much bigger in systole and diastole in relation with the first echocardiogram. The RV/LV relation changed significatively. This case describes some aspects: The importance of early detection of PH, the importance of hyperreactivity of pulmonary vasculature at early age and the positive changes in few years. RV, right ventricle; LV, left ventricle; PH, pulmonary hypertension.

In patients with a positive O_2_Test response, it was frequent to find positive changes in the clinical findings, in the echocardiogram, and sometimes in the chest X-ray after the test. In the follow-up, a positive O_2_Test correlated with the clinical outcome and paraclinical evolution, as illustrated in Figures 3 and 4.

In subgroup B1 (*n* = 10), composed of nonresponders moving to a low altitude, four patients improved their FC without experiencing a change in PASP, and one patient worsened. The five patients who died in this subgroup had severe Idiopathic PAH. On the other hand, in subgroup B2 (*n* = 16), which consists of patients with a negative O_2_Test response who continued to live at a high altitude, four patients died, eight worsened, and four are currently stable with high PASP.

The median follow-up was 7 years (IQR: 4–14) for patients with a positive O_2_Test and 3.5 years (IQR: 2–8.5) when the test was negative (*p* = 0.09).

### Mortality

**FIG. 5.**
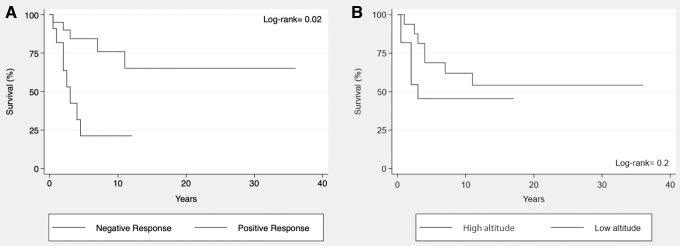
Kaplan–Meier analysis. **(A)** Kaplan–Meier survival analysis related with the response to prolonged hyperoxia test. Patients with positive PHT show longer survival with significant difference than patients with negative PHT. **(B)** Kaplan–Meier survival analysis according to whether or not they went to live at low altitude. There is not a significant difference; however, the FC is better in low altitude.

Cohort median survival time was 11 years with overall deaths secondary to PH at 23% (*n* = 12) during follow-up, one death was not related to PH. Regarding O_2_Test response, 4 patients (30.76%) died among the patients with a positive response, compared with 9 patients (69.24%) who died within the negative response group (*p* = 0.03). Kaplan–Meier survival estimates showed better long-term survival for patients with a positive O_2_Test response (Fig. 5A). Moreover, no differences were observed in survival estimates between patients who did go to live at a low altitude versus those who did not (Fig. 5B).

In the patients who died, severe pulmonary vascular disease was found at autopsy.

### Pressures and resistances

Concerning the levels of pressures and resistances found in the catheterization, the differences were statistically significant in the median of the systolic pulmonary pressure (88 mm Hg vs. 110 mm Hg; *p* = 0.038), diastolic pulmonary pressure (37 mm Hg vs. 56 mm Hg; *p* = 0.035), and in the mean pulmonary pressure (57 mm Hg vs. 88 mm Hg; *p* = 0.038) according to the positive or negative response to the O_2_Test, respectively. The difference in the median of the pulmonary resistances was not statistically significant according to the positive or negative response to O_2_Test (18 vs. 23 wood unit (WU), respectively; *p* = 0.25).

There were differences in pulmonary artery pressure and resistances if the diagnosis was made before or after 3 years of age, but these differences were not statistically significant.

Finally, there were significant differences in pulmonary artery pressure and resistances at diagnosis between patients that are still alive versus those who have died during the follow-up (*p* = 0.002).

Table 2 shows the PASP estimated by echocardiography according to the response to the O_2_Test. In the patients with a positive response, there was a statistically significant difference in the mean of PASP before and after O_2_Test (*p* = 0.001).

**Table 2.** Pulmonary Systolic Pressure (Mean and Range in mm Hg) Before and After the O_2_Test, According to the Response to the Test

O_2_Test	Basal	After test	Range	*p*
Positive	110.2	63.5	80–160	0.001
Negative	117.25	109.9	68–168	

There was not a significant difference in the FC or survival according to the presence or absence of foramen ovale or ASD; however, the four patients with a defect >5 mm in diameter are alive in good FC (FC 2).

## Discussion

Most of the studies in PH have been done in adults and at sea level, and their results have been extrapolated to the pediatric population (including children who live at high altitudes), without considering the effect of hypobaric hypoxia related to altitude (Díaz and Márquez, 2011; Rajagopal et al., 2016). It is important to recognize that children with PH living at high altitudes are different than children with PH living at low altitudes or at sea level, especially regarding the hyperreactivity of the pulmonary vasculature, as has been identified, even in healthy newborns, in studies performed in Peru (Penaloza et al., 1963; Sime et al., 1963; Gamboa and Marticorena, 1971). In one study, we found a delayed reduction of pulmonary artery pressure after birth in Bogotá in 10.45% of 95 healthy newborns (Jácome Lobo et al., 2017).

There is little knowledge about the characteristics of PH in children living at a high altitude. There have been some studies developed at very high altitudes (Pollard et al., 2001; Penaloza et al., 2008); however, the majority of people living at high altitudes live between 2,500 and 4,000 MASL, and there have been few studies conducted at this average high altitude level (Khoury and Hawes, 1963; Fasules et al., 1985; Díaz and Márquez, 2011). The classical anatomopathological study on primary PH by Wagenvoort and Wagenvoort (1970) included mainly adults. It is unknown how many of these patients were children when the disease began, considering that PH initially is a “silent disease.” In fact, in most of our children with severe PH, the initial unique findings were fatigue or syncope and some only had heart hyperactivity, and loud second heart sound.

Two factors play a pivotal role in PH, determining prognosis and therapeutic approaches: the reactivity of the pulmonary vasculature, and the remodeling of the pulmonary vasculature (Heath and Williams, 1991–1992; Haworth, 1988; Robbins et al., 2001; Stenmark and McMurtry, 2005; Díaz and Márquez, 2011; Wilkins et al., 2015; Neupane and Swenson, 2017). Furthermore, hypoxia needs to be considered when approaching a patient with PH due to its modifying effect on vascular remodeling, which is a complication to avoid. In our patients, when PH was detected early in childhood, the component of HPV (Swenson, 2013) was important, and the response to treatment and outcomes were better, confirming the importance of the reactivity test and early detection of PH, as illustrated in Figures 3 and 4.

Considering the effect of hypobaric hypoxia on patients with PH at high altitude, the response to oxygen has a special significance to evaluate the reactivity at these acute altitudes. We designed the O_2_Test to detect and evaluate the role of hypoxia at high altitude. HPV is more frequent at higher altitudes, more significant in children, and more noticeable the younger the children are (Figs. 3 and 4). This characteristic varies from person to person and among different species (Grover et al., 1963). HPV has been known since the classic article by Paul Wood (1958) and before (Swenson, 2013).

The proposed O_2_Test allowed us to rescue patients with severe PH in whom the catheterization with conventional reactivity tests did not provide any possibility of survival, as was the case with patient 9 (Table 1 and Fig. 3), or patient 8, who was initially rejected for surgery. The O_2_Test also helped to evaluate prognosis as is illustrated with the patient in Figure 4 who had severe PH and tiny left ventricle, allowing to identify that the patient had few remodeling as has been confirmed in the long follow up, the current fuctional class (FC1) and the level of BNP. For the same reasons, during catheterization in the vascular reactivity test in children at high altitude, we use, not only nitric oxide (NO), but also hyperoxia. Different to sea level, in a Cath Lab for vascular reactivity test, we recommend to use initially hyperoxia with FIO_2_ >80 (as in O_2_Test) and after, NO with the standard parameters by the effect of hypobaric hypoxia.

Some aspects of our data are relevant. When the diagnosis was made at 3 years old or younger, out of 23 patients, the O_2_Test was positive in 16 (69.6%) of them, whereas in patients over 3 years of age, only 10 out of 29 (34.48%) were positive (*p* = 0.014). This means that younger patients had more vasoreactivity and less remodeling, which confirms the importance of early detection of PH (Díaz and Márquez, 2011; Humbert et al., 2012). The FC was significantly better in patients with positive O_2_ Test, correlating with the finding that there were fewer deaths among positive responders to the O_2_Test (30.7% vs. 69.3%, *p* = 0.03).

In summary, this test allows us to identify patients, who although having severe PH, this high pressure is secondary, at less partially, to hyperreactivity of the pulmonary vasculature, influenced by hypobaric hypoxia (pulmonary vasoconstriction) without having important remodeling. This concept is clearly demonstrated with the evolution of patients of Figures 3 and 4. In the future, it could be interesting to reevaluate with O2Test the patients who continue to live in Bogotá, comparing them with patients that went to live at low altitude; however, this would be a new study.

There was a significant difference in survival according to the response to the O_2_Test, being longer when the response to the test was positive (Fig. 5A). However, there was not a significant difference in survival between patients who went to live at a low altitude and patients who continued living at a high altitude (Fig. 5B).

However, considering the significant difference in FC (is better in patients living at low altitude) and also the follow-up, in the future, the difference in survival could be significant, as is illustrated with patients with a longer follow-up: median of 30.6 years (range = 18–37 years). They had a positive O_2_Test, were diagnosed at an early age, and went to live at a low altitude very soon after the diagnosis of PH (Table 1). These patients were in FC 3 (very bad) at diagnosis (one of them, had systemic pulmonary artery pressure and grade II histological changes) (Heath and Edwards, 1958), and at this moment of follow-up, are asymptomatic with normal pulmonary artery pressure or mild PH (Grover et al., 1966). One of them (Fig. 3) has a BMPR2 mutation.

We must differentiate between the treatment of PH and a therapeutic approach to PH. The best therapeutic approach for patients with PH is early detection to avoid the remodeling of pulmonary vasculature. Regarding the treatment of children with PH living at high altitudes, all of them (with positive or negative O_2_Test) must relocate to a low altitude (Khoury and Hawes, 1963; Sime et al., 1971; Díaz and Márquez, 2011) and use vasodilators (Aldashev et al., 2005; Kojonazarov et al., 2012). We do not use calcium channel blockers because we have observed excellent results in PH in children living at high altitudes with sildenafil, including patients with persistent PH of the newborn.

We found better outcomes, better FC, and better quality of life in patients who went to live at low altitudes (Fig. 2) in both responders and nonresponders; however, the response in both groups was different. In the responder group, patients who relocated to a low altitude (Subgroup A1) experienced significant clinical improvement and an important lowering of the pulmonary pressure. On the other hand, in the nonresponder group, patients who went to live at a low altitude (Subgroup B1), improved their FC, but the pulmonary artery pressure did not change or increase. On the contrary, in both subgroups of patients who could not go to live at a low altitude (A2 and B2), the prognosis was poor and they died or their condition worsened. These outcomes support the importance of living at a low altitude for patients with PH.

It is noticeable that of the two patients that were diagnosed in Bogotá but were born at low altitude, one died very soon, and the other one is in FC 3 although both returned to live at low altitude after the diagnosis of PH.

Until recently, the prognosis of idiopathic pulmonary hypertension was poor (D'Alonzo et al., 1991; Widlitz and Barst, 2003). In our patients, the median age at diagnosis was 4.5 years, the median of the follow-up lasted 4.5 years (range: 0.5–37 years), and several of the patients are presently asymptomatic living at low altitudes, mainly when the diagnosis was made at 3 years of age or younger.

There are limitations to our study. The sample size was small (although is the largest at this altitude) and not all children could be studied (One patient had not tricuspid regurgitation). Furthermore, it is known that evaluation of PH by echocardiography gives an estimate of PASP. We tried to standardize the oxygen protocol but this was difficult due to different availability of equipment. However the principle is to supply FiO_2_ >80%.

## Conclusion

Considering the effect of hypobaric hypoxia, children with PH living at high altitudes need a specific diagnostic approach to evaluate hypoxic vasoconstriction, which is important in the pathogenesis of PH and is notorious at an early age. This explains the importance of early detection of PH to avoid the remodeling of pulmonary vasculature; although at high altitudes it is frequent to find severe PH at an early age.

The proposed O_2_Test through echocardiography allows detection of patients who, although having severe PH, also have hyperreactivity of pulmonary vasculature. Patients with a positive O_2_Test have better FC and better prognosis than children with a negative O_2_Test.

The outcome of patients with a positive response to the O_2_Test who relocate to a low altitude (with vasodilators) is better and some of them could become asymptomatic if diagnosed at an early age. The nonresponders if relocated to a low altitude have better quality of life and better survival rates.
